# Anterior Cruciate Ligament Reconstruction Surgery Outcomes: A Comparison Between Patients Who Underwent the Procedure During the COVID-19 Pandemic and a Cohort Treated Prior to the Pandemic

**DOI:** 10.7759/cureus.57840

**Published:** 2024-04-08

**Authors:** Trevor D Wolterink, Michael A Gaudiani, Rami S Beydoun, Johnny K Kasto, Ryan Y Sanii, Vasilios Moutzouros, Stephanie Muh

**Affiliations:** 1 Department of Orthopaedic Surgery, Wayne State University School of Medicine, Detroit, USA; 2 Department of Orthopaedic Surgery, Henry Ford Health System, Detroit, USA; 3 Department of Orthopaedic Surgery, Corewell Health East, Royal Oak, USA; 4 Department of Orthopaedic Surgery, Michigan State University College of Human Medicine, Lansing, USA

**Keywords:** knee arthroscopy, anterior cruciate ligament (acl), knee ligaments, injuries, covid-19 pandemic, knee

## Abstract

Background and objective

During the coronavirus disease 2019 (COVID-19) pandemic, many elective orthopedic surgeries, including anterior cruciate ligament reconstruction (ACLR), were temporarily postponed. The purpose of this study was to compare the outcomes of ACLR in patients who underwent surgery during the COVID-19 pandemic with those in a cohort treated before the pandemic.

Materials and methods

This retrospective review compared patients who underwent primary ACLR during two periods: March to June 2020 (the pandemic group) and January to December 2018 (the pre-pandemic group). Matched cohorts (1:1) were created using propensity matching. Time from injury-to-first visit, injury-to-surgery, and first visit-to-surgery were calculated. Subjective and objective outcomes, minimal clinically important difference (MCID) achievement, and complication rates were recorded for up to two years postoperatively. Statistical analysis included 𝛘2 or Fisher’s exact tests for categorical data, and t- or Wilcoxon signed-rank tests for continuous data with significance set at *P *< 0.05.

Results

The pandemic and pre-pandemic groups consisted of 33 and 217 patients, respectively. Matched cohorts consisted of 33 patients each. The time from injury-to-surgery and the first visit-to-surgery was prolonged in the pandemic group. When unmatched, visual analog scale (VAS) scores at three months postoperatively and Patient-Reported Outcomes Measurement Information System (PROMIS)-pain interference (PI) at six months postoperatively and at the final follow-up were higher in the pandemic group. When matched, PROMIS-PI at six months postoperatively was higher in the pandemic group, and VAS scores at one year postoperatively were higher in the pre-pandemic group. MCID achievement and complication rates did not significantly differ between the groups.

Conclusions

ACLR procedures were significantly delayed in the early months of the COVID-19 pandemic. While patients treated before and during the pandemic experienced varying pain levels during recovery, their functional outcomes, MCID achievement, and complication rates did not differ significantly.

## Introduction

The first cases of severe acute respiratory syndrome coronavirus 2 (SARS-CoV-2) infection were reported in Wuhan, China in December 2019 [1], and the virus spread globally in the next three months. Coronavirus disease 2019 (COVID-19) was declared a pandemic by the World Health Organization, and stay-at-home orders were issued across the United States [2]. The national healthcare system responded with reorganization measures to stem the spread of the disease and conserve limited healthcare resources. As part of this response, an executive order was issued in Michigan, requiring the postponing or canceling of elective surgeries [3]. The interruption in surgical procedures imposed by this decision likely had a significant impact on the field of orthopedic surgery, which witnessed over 4.8 million elective procedures in the United States in 2017 alone [4]. In addition, the orthopedic procedures that continued to be offered during this period were affected by unprecedented disruption to the healthcare system and society more broadly. Preliminary research suggests that patients who underwent surgery during the COVID-19 pandemic experienced an increase in delays and complications, but the outcomes of the surgeries performed at the height of this period remain largely unknown [5-8].

Anterior cruciate ligament (ACL) tears are common knee injuries, with an estimated incidence of 100,000 to 200,000 per year in the United States [9]. ACL reconstruction (ACLR) to stabilize the knee is currently considered the gold standard treatment as trends have shown better functional outcomes after surgery [10]. Several factors emerged during the pandemic that may have impacted the outcomes after ACLR. These included surgical delays, disrupted patient follow-up, and reduced access to rehabilitation facilities as well as patient-specific factors, such as a shift to more sedentary lifestyles due to quarantine protocols [11,12]. The pandemic, therefore, presents a unique opportunity to assess patients who experienced a disrupted standard of care following an ACL tear and evaluate their postoperative outcomes.

In light of this, this retrospective cohort study aimed to compare outcomes for patients who underwent ACLR at a single institution at the height of the COVID-19 pandemic with those of a pre-pandemic cohort. We hypothesized that the COVID-19 pandemic cohort experienced prolonged time-to-surgery, poorer functional outcomes, and a higher incidence of surgical complications and reoperations.

## Materials and methods

**Figure 1:**
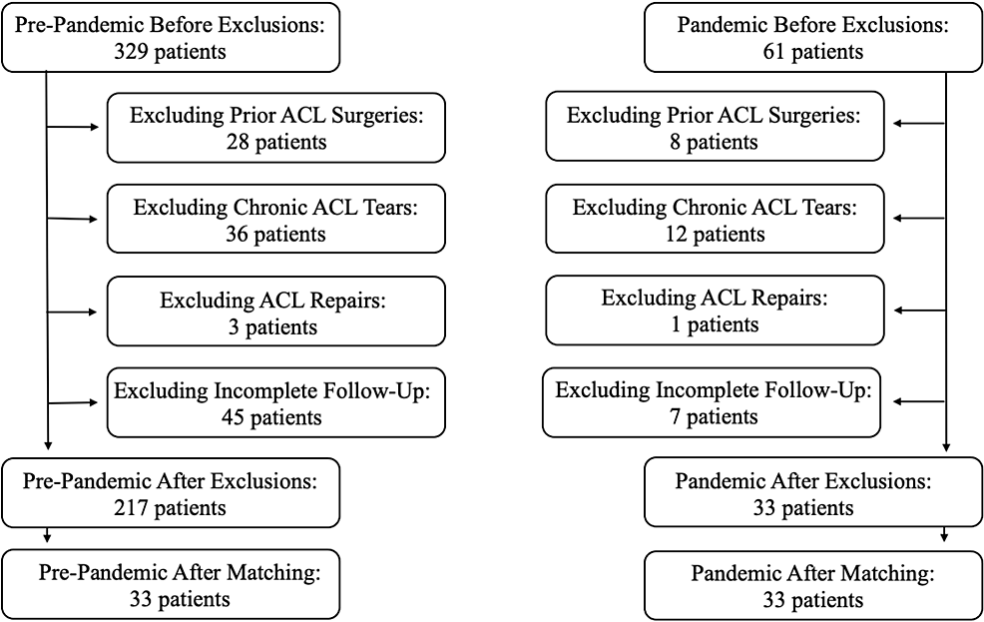
Flowchart Depicting the Exclusion of Patients The pre-pandemic group exclusion flowchart is displayed on the left, and the pandemic group exclusion flowchart is displayed on the right. The number of patients prior to exclusions is shown at the top of the figure with the number of patients excluded for each criterion shown below. The pre-pandemic group consisted of 217 patients after exclusions and 33 patients after propensity matching. The pandemic group consisted of 33 patients after exclusions and 33 patients after propensity matching ACL: anterior cruciate ligament

After obtaining institutional review board approval, we performed a retrospective review of patients who underwent ACLR by 12 board-certified orthopedic surgeons in a single healthcare system. Patients were identified by querying a billing record database for Current Procedural Terminology code 29888. Two cohorts were created based on the date of surgery. The pre-pandemic cohort consisted of patients who underwent ACLR between January to December of 2018 (the pre-pandemic group); the COVID-19 pandemic cohort comprised patients who underwent surgery between March and June of 2020 (the pandemic group) [2,3]. Patients with prior ACL surgery or chronic ACL tears (greater than six months before surgery), those who underwent ACL repair, and patients with incomplete follow-up at 3, 6, and 12 months postoperatively were excluded from the study (Figure 1).

A retrospective chart review was conducted to obtain demographic information, injury characteristics, baseline function, and outcomes after surgery. Demographic-related data included age, sex, race, BMI, history of diabetes mellitus, smoking status, marital status, and healthcare insurance provider. Time from the injury-to-first visit and injury-to-surgery were calculated as the number of days from the date of the injury to the date of the first encounter with the surgeon or the date of the surgery, respectively. Time from the first visit-to-surgery was calculated as the number of days from the first encounter with the surgeon to the date of the surgery. The laterality of the ACL injury was noted. MRI findings recorded in the preoperative note of the orthopedic surgeon who performed the surgery were reviewed to determine whether the injury was associated with a meniscal tear or an additional ligamentous tear of the knee. Data on surgery duration and type of graft used for reconstruction were gathered from the operative note. 

Visual analog scale (VAS) pain scores and Patient-Reported Outcomes Measurement Information System (PROMIS) physical function (PF), pain interference (PI), and depression (D) measures as well as knee range of motion and ACL tests, including Lachman, pivot shift, and anterior drawer tests, were recorded preoperatively; at 3-, 6-, and 12-months postoperatively; and at the final visit within 2 years of surgery. VAS pain score was measured on a scale of 0 to 10. Differences in PROMIS-PF and PROMIS-PI were calculated from the preoperative to final visit time interval. MCID was determined from a previous study using the anchor-based methodology and found to be +4.5 for PROMIS-PF, -5.4 for PROMIS-PI, and -4.1 for PROMIS-D [13]. Physical examination information was recorded at clinic visits and collected from notes in the medical record. Lachman test was considered negative in cases of Grade 1A and positive in Grade 1B or higher. Pivot shift and anterior drawer tests were recorded as positive or negative. Physical therapy notes were reviewed to determine the setting of therapy (in-person, virtual, mixed) and the number of visits. All relevant medical records were reviewed up to two years postoperatively to determine whether a complication or revision surgery occurred. Statistical analysis was performed to compare the unmatched pre-pandemic and pandemic cohorts.

Propensity matching for age, sex, race, BMI, and smoking status was performed, and 1:1 matched pre-pandemic and pandemic groups were created. Statistical analysis was performed to compare demographic information, injury characteristics, baseline function, and outcomes after surgery between the matched pre-pandemic and pandemic cohorts.

Statistical analysis

All analyses were performed using SPSS Statistics 28.0.1.1 (IBM, Armonk, NY). Descriptive statistics were calculated with categorical data reported as counts with percentages and continuous data as mean ± standard deviation (SD). To evaluate categorical data, two-group univariate comparisons were analyzed using 𝛘2 for values greater than 5 and Fisher's exact tests for values less than 5. Continuous variables were analyzed using a two-sample t-test for normally distributed variables and Wilcoxon signed-rank for non-normally distributed variables. Statistical significance was set at *P *< 0.05.

## Results

A total of 250 patients met the inclusion criteria and were included in our study; 217 of them underwent ACLR in 2018 (the pre-pandemic group) compared to 33 between March and June 2020 (the pandemic group). After propensity matching, 33 patients each were included in both the pre-pandemic and pandemic groups (Figure 1). Lack of follow-up as a reason for exclusion was not significantly different between the cohorts (13.7% for pre-pandemic vs 11.5% for pandemic, *P *= 0.42).

Comparison: unmatched pre-pandemic and pandemic groups

The groups significantly differed in terms of sex, with a higher percentage of males in the pre-pandemic group (55.3% vs 30.3%, *P *= 0.01). No other significant demographic differences were observed between the cohorts (Table 1).

**Table 1:** Comparison Between Unmatched Pre-Pandemic and Pandemic Groups: Demographics ^a^Indicates statistically significant difference at *P *< 0.05 Percentages rounded to the nearest 10th and may not equate to 100% SD: standard deviation

Variable	Pre-Pandemic Group (N = 217)	Pandemic Group (N = 33)	*P*-value
Age (years), mean ± SD	25.7 ± 10.7	26.8 ± 11.0	0.559
Sex, n (%)			0.009^a^
Female	97 (44.7)	23 (69.7)	
Male	120 (55.3)	10 (30.3)	
Race, n (%)			0.301
Caucasian	147 (67.7)	18 (54.5)	
African American	33 (15.2)	8 (24.2)	
Other	37 (17.1)	7 (21.2)	
Body mass index (kg/m^2^), mean ± SD	27.0 ± 6.0	27.3 ± 7.5	0.615
Diabetes mellitus, n (%)			1.000
Yes	3 (1.4)	0 (0)	
No	214 (98.6)	33 (100)	
Current smoker, n (%)			1.000
Yes	26 (12.0)	4 (12.1)	
No	191 (88.0)	29 (87.9)	
Marital status, n (%)			0.207
Single	166 (76.5)	29 (87.9)	
Married	46 (21.2)	3 (9.1)	
Other	5 (2.3)	1 (3.0)	
Insurance type, n (%)			0.722
Medicaid/Medicare	28 (12.9)	3 (9.1)	
Private	180 (82.9)	28 (84.8)	
Worker’s compensation	9 (4.1)	2 (6.1)	

Time from injury-to-first visit was not significantly different between the groups (22.0 ± 23.2 days for pre-pandemic vs 28.2 ± 27.4 days for pandemic, *P *= 0.23). Time from injury-to-surgery (55.0 ± 32.6 days vs 83.4 ± 38.5 days, *P *< 0.01) and first visit-to-surgery (33.0 ± 23.8 days vs 55.2 ± 43.0 days, *P *= 0.01) were significantly prolonged in the pandemic cohort.

Sports played during an injury were significantly different between the groups (*P *= 0.04). Basketball was the most common sport played during an injury in both cohorts (19.8% for pre-pandemic vs 24.2% for pandemic) followed by soccer in the pre-pandemic cohort (18.9% vs 12.1%) and skiing in the pandemic cohort (5.1% vs 21.2%). Injury mechanism, laterality of ACL tear, presence of a concomitant meniscal or multi-ligamentous tear, and surgery duration were not significantly different between the groups (Table 2). Graft type was significantly different between the cohorts (*P *< 0.01). Bone-patellar tendon-bone autograft was the most common graft type in both groups (53.0% vs 54.5%) followed by hamstring autograft in the pre-pandemic group (25.8% vs 9.1%) and quadriceps autograft in the pandemic group (3.7% vs 30.3%). Injury and treatment characteristics are displayed in Table 2.

**Table 2:** Comparison Between Unmatched Pre-Pandemic and Pandemic Groups: Injury and Treatment Characteristics ^a^Indicates statistically significant difference at *P* < 0.05 Percentages rounded to the nearest 10th and may not equate to 100% BTB: bone-patellar tendon-bone; SD: standard deviation

Variable	Pre-Pandemic Group (N = 217)	Pandemic Group (N = 33)	P-value
Injury-to-first visit (days), mean ± SD	22.0 ± 23.2	28.2 ± 27.4	0.228
Injury-to-surgery (days), mean ± SD	55.0 ± 32.6	83.4 ± 38.5	<0.001^a^
First visit-to-surgery (days), mean ± SD	33.0 ± 23.8	55.2 ± 43.0	0.012^a^
Injury sport, n (%)			0.036^a^
Basketball	43 (19.8)	8 (24.2)	
Football	23 (10.6)	0 (0)	
Skiing	11 (5.1)	7 (21.2)	
Soccer	41 (18.9)	4 (12.1)	
Softball	7 (3.2)	1 (3.0)	
Volleyball	8 (3.7)	0 (0)	
Other sport	33 (15.2)	5 (15.2)	
No sport	36 (16.6)	4 (12.1)	
Unspecified	15 (6.9)	4 (12.1)	
Injury mechanism, n (%)			0.299
Atraumatic, unspecified	28 (12.9)	5 (15.2)	
Traumatic	79 (36.4)	11 (33.3)	
Cut, pivot, or twist	36 (16.6)	7 (21.2)	
Jump or plant	54 (24.9)	4 (12.1)	
Hyperextension	6 (2.8)	1 (3.0)	
Unspecified	14 (6.5)	5 (15.2)	
Laterality, n (%)			0.348
Right	121 (55.8)	15 (45.5)	
Left	96 (44.2)	18 (54.5)	
Meniscal tear, n (%)			1.000
Yes	139 (64.1)	21 (63.6)	
No	78 (35.9)	12 (36.4)	
Multi-ligamentous tear, n (%)			0.314
Yes	38 (17.5)	3 (9.1)	
No	179 (82.5)	30 (90.9)	
Surgery duration (minutes), mean ± SD	93.1 ± 46.6	97.3 ± 53.4	0.970
Graft type, n (%)			<0.001^a^
BTB autograft	115 (53.0)	18 (54.5)	
BTB allograft	19 (8.8)	0 (0)	
Hamstring autograft	56 (25.8)	3 (9.1)	
Quadriceps autograft	8 (3.7)	10 (30.3)	
Other graft	19 (8.8)	2 (6.1)	

VAS scores (3.8 ± 2.6 for pre-pandemic vs 3.6 ± 2.3 for pandemic, *P *= 0.86) and PROMIS-PF (36.4 ± 9.1 vs 37.1 ± 7.5, *P *= 0.60), PROMIS-PI (61.9 ± 5.9 vs 59.5 ± 7.1, *P *= 0.35), and PROMIS-D (48.8 ± 9.3 vs 50.1 ± 8.8, *P *= 0.61) were not significantly different between groups preoperatively. However, VAS scores were significantly higher in the pandemic cohort at three months postoperatively (1.2 ± 1.8 vs 1.9 ± 2.1, *P *= 0.02). There were no other differences in VAS scores between groups up to two years postoperatively (Table 3). PROMIS-PI was significantly higher in the pandemic cohort at six months postoperatively (47.9 ± 7.3 vs 52.4 ± 7.9, *P *= 0.02) and final follow-up (45.2 ± 8.2 vs 53.2 ± 8.9, *P *= 0.01). There were no significant differences in PROMIS-PF or PROMIS-D between groups at two years postoperatively (Table 3). Achievement of MCID for PROMIS-PF (87.5% vs 100%, *P *= 1.00) and PROMIS-PI (93.8% vs 66.7%, *P *= 0.30) at final follow-up was not significantly different between cohorts. Knee range of motion in flexion and extension and ACL tests were not significantly different between groups up to 2 years postoperatively (Table 3). Outcome data are summarized in Tables 3-4.

**Table 3:** Comparison Between Unmatched Pre-Pandemic and Pandemic Groups: Outcome Characteristics ^a^Indicates statistically significant difference at *P* < 0.05 D: depression; PF: physical function; PI: physical interference; PROMIS: Patient-Reported Outcomes Measurement Information System; SD: standard deviation; VAS: visual analog scale

Variable	Pre-Pandemic Group (N = 217), Mean ± SD	Pandemic Group (N = 33), Mean ± SD	*P-*value
Preoperative	N = 217	N = 33	
Days to surgery	22.5 ± 18.2	36.5 ± 33.7	0.130
VAS	3.8 ± 2.6	3.6 ± 2.3	0.856
PROMIS-PF	37.1 ± 9.1	37.1 ± 7.5	0.596
PROMIS-PI	61.9 ± 5.9	59.5 ± 7.1	0.350
PROMIS-D	48.8 ± 9.3	50.1 ± 8.8	0.611
Knee extension, degrees	4.5 ± 7.0	2.3 ± 4.4	0.181
Knee flexion, degrees	108.4 ± 22.9	110.6 ± 18.7	0.859
3 months	N = 205	N = 29	
Days from surgery	86.6 ± 16.8	89.8 ± 18.8	0.347
VAS	1.2 ± 1.8	1.9 ± 2.1	0.022^a ^
PROMIS-PF	44.1 ± 4.7	46.8 ± 9.9	0.693
PROMIS-PI	52.4 ± 7.3	56.4 ± 7.6	0.083
PROMIS-D	42.1 ± 9.8	47.0 ± 10.6	0.286
Knee extension, degrees	0.6 ± 2.1	0.8 ± 1.9	0.862
Knee flexion, degrees	122.8 ± 11.2	123.6 ± 9.2	0.912
6 months	N = 166	N = 27	
Days to surgery	177.3 ± 22.2	183.4 ± 19.2	0.131
VAS	0.8 ± 1.6	1.4 ± 2.4	0.279
PROMIS-PF	51.8 ± 8.1	50.6 ± 10.9	0.562
PROMIS-PI	47.9 ± 7.3	52.4 ± 7.9	0.018^a^
PROMIS-D	40.3 ± 7.4	48.3 ± 13.6	0.483
Knee extension, degrees	0.3 ± 1.5	0.2 ± 0.9	0.875
Knee flexion, degrees	127.6 ± 8.4	127.7 ± 8.6	0.872
12 months	N = 74	N = 4	
Days to surgery	362.0 ± 17.2	371.8 ± 22.4	0.452
VAS	1.8 ± 2.2	0.3 ± 0.5	0.212
PROMIS-PF	57.1 ± 10.3	55.3 ± 11.8	0.445
PROMIS-PI	47.8 ± 8.4	49.7 ± 9.2	0.262
PROMIS-D	40.7 ± 9.3		
Knee extension, degrees	0.4 ± 1.2	0.5 ± 1.0	0.314
Knee flexion, degrees	126.8 ± 10.3	132.5 ± 2.9	0.193
Final visit	N = 34	N = 12	
Days to surgery	514.2 ± 98.1	541.1 ± 130.5	0.774
VAS	1.7 ± 2.3	2.8 ± 3.3	0.617
PROMIS-PF	56.4 ± 9.6	53.9 ± 15.5	0.672
PROMIS-PI	45.2 ± 8.2	53.2 ± 8.9	0.007^a^
PROMIS-D	42.0 ± 9.1		
Knee extension, degrees	0.6 ± 2.0	0.8 ± 1.8	0.749
Knee flexion, degrees	128.0 ± 9.9	126.9 ± 12.2	0.984

**Table 4:** Comparison Between Unmatched Pre-Pandemic and Pandemic Groups: MCID Achievement at Final Visit Percentages rounded to the nearest 10th and may not equate to 100% MCID: minimal clinically important difference; PF: physical function; PI: physical interference; PROMIS: Patient-Reported Outcomes Measurement Information System; SD: standard deviation

Variable	Pre-Pandemic Group (N = 217), N (%)	Pandemic Group (N = 33), N (%)	*P-*value
PROMIS-PF	N = 16	N = 3	1.000
Yes	14 (87.5)	3 (100)	
No	2 (12.5)	0 (0)	
PROMIS-PI	N = 16	N = 3	0.298
Yes	15 (93.8)	2 (66.7)	
No	1 (6.3)	1 (33.3)	

Attendance of physical therapy was not significantly different between the cohorts (99.5% for pre-pandemic vs 97.0% for pandemic, *P* = 0.25). Also, the type of physical therapy was not significantly different between groups (*P* = 0.23). In-person physical therapy was the most common type of physical therapy in both cohorts (99.3% vs 95.0%). One patient in the pre-pandemic group attended mixed in-person and virtual physical therapy, and one patient in the pandemic group attended virtual physical therapy. The number of physical therapy visits was not significantly different between cohorts (26.2 ± 13.7 vs 31.0 ± 22.5, *P* = 0.37). Physical therapy characteristics are displayed in Table 5.

**Table 5:** Comparison Between Unmatched Pre-Pandemic and Pandemic Groups: Physical Therapy Characteristics Percentages rounded to the nearest 10th and may not equate to 100% SD: standard deviation

Variable	Pre-Pandemic Group (N = 217)	Pandemic Group (N = 33)	*P-*value
Attended? n (%)	N = 217	N = 33	0.247
Yes	216 (99.5)	32 (97.0)	
No	0 (0)	1 (3.0)	
Unspecified	1 (0.5)	0 (0)	
Type, n (%)	N = 141	N = 20	0.234
In-person	140 (99.3)	19 (95.0)	
Virtual	0 (0)	0 (0)	
Mixed	1 (0.7)	1 (5.0)	
Number of visits, mean ± SD	26.2 ± 13.7	31.0 ± 22.5	0.365

The complication rate was not significantly different between the groups (15.7% for pre-pandemic vs 9.1% for pandemic, *P* = 0.43). A meniscal tear was the most common complication in both cohorts (6.9% vs 6.1%). The secondary surgery rate was not significantly different between the groups (9.2% vs 9.1%, *P* = 1.00). Manipulation, lysis, or debridement was the most common secondary surgery in the pre-pandemic cohort (4.6% vs 3.0%), while meniscus repair or meniscectomy was the most common procedure in the pandemic cohort (3.2% vs 6.1%). The rate of ACL graft failure was 4.1% in the pre-pandemic group and 3.3% in the pandemic group. Complication-related data are summarized in Table 6.

**Table 6:** Comparison Between Unmatched Pre-Pandemic and Pandemic Groups: Complication Characteristics Data shown are count (percentages). *P-*values compare complication and secondary surgery rates between the pre-pandemic and pandemic groups. Types of complications and secondary surgery were not compared between groups. Patients may have sustained or undergone more than one complication or surgery, respectively ACL: anterior cruciate ligament

Variable	Pre-Pandemic Group (N = 217)	Pandemic Group (N = 33)	*P-*value
Complication?	34 (15.7)	3 (9.1)	0.434
ACL retear	9	1	
Meniscus tear	15	2	
Stiffness	11	1	
Osteoarthritis	4	0	
Painful hardware	5	0	
Infection	1	0	
Secondary surgery?	20 (9.2)	3 (9.1)	1.000
ACL revision	6	1	
Meniscus repair or meniscectomy	7	2	
Manipulation, lysis, or debridement	10	1	
Hardware removal	5	0	
Incision and drainage	1	0	

Comparison: matched pre-pandemic and pandemic groups

There were no significant differences between the groups regarding demographics (Table 7).

**Table 7:** Comparison Between Matched Pre-Pandemic and Pandemic Groups: Demographics Percentages rounded to the nearest 10th and may not equate to 100% SD: standard deviation

Variable	Pre-Pandemic Group (N = 33)	Pandemic Group (N = 33)	*P-*value
Age (years), mean ± SD	25.1 ± 11.5	26.8 ± 11.0	0.352
Gender, n (%)			1.000
Female	23 (69.7)	23 (69.7)	
Male	10 (30.3)	10 (30.3)	
Race, n (%)			0.936
Caucasian	18 (54.5)	18 (54.5)	
African American	7 (21.2)	8 (24.2)	
Other	8 (24.2)	7 (21.2)	
Body mass index (kg/m^2^), mean ± SD	26.5 ± 4.8	27.3 ± 7.5	0.788
Diabetes mellitus			
Yes	0 (0)	0 (0)	
No	33 (100)	33 (100)	
Current smoker, n (%)			1.000
Yes	5 (15.2)	4 (12.1)	
No	28 (84.8)	29 (87.9)	
Marital status, n (%)			0.185
Single	25 (75.8)	29 (87.9)	
Married	8 (24.2)	3 (9.1)	
Other	0 (0)	1 (3.0)	
Insurance type, n (%)			1.000
Medicaid/Medicare	3 (9.1)	3 (9.1)	
Private	27 (81.8)	28 (84.8)	
Worker’s compensation	3 (9.1)	2 (6.1)	

Time from injury-to-first visit was not significantly different between the groups (23.6 ± 26.1 days for pre-pandemic vs 28.2 ± 27.4 days for pandemic, *P* = 0.61). However, time from injury-to-surgery (55.1 ± 31.2 days vs 83.4 ± 38.5 days, *P* < 0.01) and first-visit-to-surgery (31.5 ± 23.3 days vs 55.2 ± 43.0 days, *P* = 0.03) were significantly prolonged in the pandemic cohort.

Sports played during injury, injury mechanism, laterality of ACL tear, presence of a concomitant meniscal or multi-ligamentous tear, and surgery duration were not significantly different between the groups (Table 8). However, graft type was significantly different between cohorts (*P* < 0.01). Bone-patellar tendon-bone autograft was the most common graft type in both groups (42.4% for pre-pandemic vs 54.5% for pandemic) followed by hamstring autograft in the pre-pandemic group (39.4% vs 9.1%) and quadriceps autograft in the pandemic group (0% vs 30.3%). Injury and treatment characteristics are displayed in Table 8.

**Table 8:** Comparison Between Matched Pre-Pandemic and Pandemic Groups: Injury and Treatment Characteristics ^a^Indicates statistically significant difference at *P* < 0.05 Percentages rounded to the nearest 10th and may not equate to 100% BTB: bone-patellar tendon-bone; SD: standard deviation

Variable	Pre-Pandemic Group (N = 33)	Pandemic Group (N = 33)	*P-*value
Injury-to-first visit (days), mean ± SD	23.6 ± 26.1	28.2 ± 27.4	0.612
Injury-to-surgery (days), mean ± SD	55.1 ± 31.2	83.4 ± 38.5	0.001^a^
First visit-to-surgery (days), mean ± SD	31.5 ± 23.3	55.2 ± 43.0	0.032^a^
Injury sport, n (%)			0.113
Basketball	6 (18.2)	8 (24.2)	
Football	3 (9.1)	0 (0)	
Skiing	2 (6.1)	7 (21.2)	
Soccer	4 (12.1)	4 (12.1)	
Softball	1 (3.0)	1 (3.0)	
Volleyball	3 (9.1)	0 (0)	
Other sport	4 (12.1)	5 (15.2)	
No sport	9 (27.3)	4 (12.1)	
Unspecified	1 (3.0)	4 (12.1)	
Injury mechanism, n (%)			0.826
Atraumatic, unspecified	3 (9.1)	5 (15.2)	
Traumatic	13 (39.4)	11 (33.3)	
Cut, pivot, or twist	9 (27.3)	7 (21.2)	
Jump or plant	5 (15.2)	4 (12.1)	
Hyperextension	1 (3.0)	1 (3.0)	
Unspecified	2 (6.1)	5 (15.2)	
Laterality, n (%)			0.083
Right	22 (66.7)	15 (45.5)	
Left	11 (33.3)	18 (54.5)	
Meniscal tear, n (%)			1.000
Yes	21 (63.6)	21 (63.6)	
No	12 (36.4)	12 (36.4)	
Multi-ligamentous tear, n (%)			1.000
Yes	4 (12.1)	3 (9.1)	
No	29 (87.9)	30 (90.9)	
Surgery duration, (minutes), mean ± SD	87.6 ± 41.5	97.3 ± 53.4	0.667
Graft type, n (%)			<0.001^a^
BTB autograft	14 (42.4)	18 (54.5)	
BTB allograft	4 (12.1)	0 (0)	
Hamstring autograft	13 (39.4)	3 (9.1)	
Quadriceps autograft	0 (0)	10 (30.3)	
Other graft	2 (6.1)	2 (6.1)	

VAS scores (3.1 ± 2.2 for pre-pandemic vs 3.6 ± 2.3 for pandemic, *P* = 0.29), and PROMIS-PF (36.4 ± 9.1 vs 37.1 ± 7.5, *P* = 0.82), PROMIS-PI (60.6 ± 7.3 vs 59.5 ± 7.1, *P* = 0.73), and PROMIS-D (49.9 ± 11.6 vs 50.1 ± 8.8, *P* = 0.97) were not significantly different between groups preoperatively. However, VAS scores were significantly higher in the pre-pandemic cohort at one year postoperatively (1.8 ± 2.3 vs 0.3 ± 0.5, *P* = 0.03). There were no other significant differences in VAS scores between cohorts up to two years postoperatively (Table 9). PROMIS-PI was significantly higher in the pandemic cohort at six months postoperatively (46.6 ± 4.4 vs 52.4 ± 7.9, *P* = 0.02). There were no other significant differences in PROMIS-PF, PROMIS-PI, or PROMIS-D between groups up until one year postoperatively (Table 9). Knee range of motion in extension was significantly greater in the pandemic group preoperatively (4.9 ± 4.9 vs 2.3 ± 4.4, *P* = 0.03). There were no other differences in knee range of motion in flexion and extension or ACL tests up to two years postoperatively (Table 9). Outcome data are summarized in Table 9.

**Table 9:** Comparison Between Matched Pre-Pandemic and Pandemic Groups: Outcome Characteristics ^a^Indicates statistically significant difference at *P* < 0.05 D: depression; PF: physical function; PI: physical interference; PROMIS: Patient-Reported Outcomes Measurement Information System; SD: standard deviation; VAS: visual analog scale

Variable	Pre-Pandemic Group (N = 33), Mean ± SD	Pandemic Group (N = 33), Mean ± SD	*P-*value
Preoperative	N = 33	N = 33	
Days to surgery	19.5 ± 16.3	36.5 ± 33.7	0.093
VAS	3.1 ± 2.2	3.6 ± 2.3	0.290
PROMIS-PF	36.4 ± 9.1	37.1 ± 7.5	0.823
PROMIS-PI	60.6 ± 7.3	59.5 ± 7.1	0.725
PROMIS-D	49.9 ± 11.6	50.1 ± 8.8	0.965
Knee extension, degrees	4.9 ± 4.9	2.3 ± 4.4	0.033^a^
Knee flexion, degrees	112.6 ± 14.5	110.6 ± 18.7	0.644
3 months	N = 33	N = 29	
Days from surgery	90.5 ± 14.2	89.8 ± 18.8	0.879
VAS	1.3 ± 1.6	1.9 ± 2.1	0.262
PROMIS-PF	41.8 ± 5.3	46.8 ± 9.9	0.260
PROMIS-PI	54.1 ± 8.3	56.4 ± 7.6	0.507
PROMIS-D	41.5 ± 13.9	47.0 ± 10.6	0.123
Knee extension, degrees	0.6 ± 1.5	0.8 ± 1.9	0.893
Knee flexion, degrees	121.0 ± 13.4	123.6 ± 9.2	0.551
6 months	N = 28	N = 27	
Days to surgery	177.7 ± 21.4	183.4 ± 19.2	0.303
VAS	1.3 ± 1.7	1.4 ± 2.4	0.758
PROMIS-PF	54.1 ± 9.8	50.6 ± 10.9	0.407
PROMIS-PI	46.6 ± 4.4	52.4 ± 7.9	0.020^a^
PROMIS-D	37.6 ± 5.9	48.3 ± 13.6	0.088
Knee extension, degrees	0.3 ± 0.9	0.2 ± 0.9	0.661
Knee flexion, degrees	124.6 ± 7.2	127.7 ± 8.6	0.097
12 months	N = 16	N = 4	
Days to surgery	365.3 ± 17.3	371.8 ± 22.4	0.617
VAS	1.8 ± 2.3	0.3 ± 0.5	0.034^a^
PROMIS-PF	54.7 ± 6.6	55.3 ± 11.8	0.933
PROMIS-PI	49.9 ± 8.5	49.7 ± 9.2	0.973
PROMIS-D			
Knee extension, degrees	0 ± 0	0.5 ± 1.0	0.391
Knee flexion, degrees	127.5 ± 6.3	132.5 ± 2.9	0.067
Final visit	N = 5	N = 12	
Days to surgery	492.8 ± 38.7	541.1 ± 130.5	0.263
VAS	1.0 ± 1.4	2.8 ± 3.3	0.231
PROMIS-PF		53.9 ± 15.5	
PROMIS-PI		53.2 ± 8.9	
PROMIS-D			
Knee extension, degrees	0 ± 0	0.8 ± 1.8	0.182
Knee flexion, degrees	128.8 ± 7.5	126.9 ± 12.2	0.750

Attendance, type, and number of visits regarding physical therapy, as well as complication and secondary surgery rates, were not significantly different between the groups up to 2 years postoperatively (Tables 10-11).

**Table 10:** Comparison Between Matched Pre-Pandemic and Pandemic Groups: Physical Therapy Characteristics Percentages rounded to the nearest 10th and may not equate to 100% SD: standard deviation

Variable	Pre-Pandemic Group (N = 33)	Pandemic Group (N = 33)	*P-*value
Attended? n (%)	N = 33	N = 33	1.000
Yes	33 (100)	32 (97.0)	
No	0 (0)	1 (3.0)	
Unspecified	0 (0)	0 (0)	
Type, n (%)	N = 21	N = 20	0.488
In-person	21 (100)	19 (95.0)	
Virtual	0 (0)	0 (0)	
Mixed	0 (0)	1 (5.0)	
Number of visits	27.0 ± 13.8	31.0 ± 22.5	0.824

**Table 11:** Comparison Between Matched Pre-Pandemic and Pandemic Groups: Complication Characteristics Data shown are count (percentages). *P*-values compare complication and secondary surgery rates between the pre-pandemic and pandemic groups. Types of complications and secondary surgery were not compared between groups. Patients may have sustained or undergone more than one complication or surgery, respectively ACL: anterior cruciate ligament

Variable	Pre-Pandemic Group (N = 33)	Pandemic Group (N = 33)	*P-*value
Complication?	6 (18.2)	3 (9.1)	0.475
ACL retear	3	1	
Meniscus tear	4	2	
Stiffness	1	1	
Osteoarthritis	0	0	
Painful hardware	1	0	
Infection	0	0	
Secondary surgery?	5 (15.2)	3 (9.1)	0.708
ACL revision	3	1	
Meniscus repair or meniscectomy	3	2	
Manipulation, lysis, or debridement	1	1	
Hardware removal	1	0	
Incision and drainage	0	0	

## Discussion

The most important findings of the present study were that the time from injury-to-surgery and first-visit-to-surgery were significantly prolonged during the COVID-19 pandemic. When unmatched, VAS scores at three months postoperatively and PROMIS-PI at six months postoperatively and final follow-up were significantly higher in patients who underwent surgery during the pandemic. When matched, PROMIS-PI at six months was significantly higher in pandemic patients, while VAS scores at one year were significantly higher in pre-pandemic patients. Achievement of MCID for PROMIS-PI at the final follow-up, however, was not significantly different between the groups. The complication rate was not significantly different in patients who underwent ACLR during the pandemic compared to before.

Several studies have investigated the impact of early versus delayed timing on functional outcomes after ACLR and found no significant difference in short- or long-term knee range of motion, strength, function, or stability [14-16]. In a meta-analysis including 11 randomized controlled trials and 972 patients, Shen et al. demonstrated that postoperative knee range of motion and laxity was not significantly different between early (8 days to 10 weeks) and delayed (four weeks to three months) surgery [17]. Lu et al., in a retrospective cohort study including 416 patients, found that ACLR after six months from injury was associated with inferior patient-reported outcome measures and reduced likelihood of achieving clinically significant outcomes [18]. In our study, the pandemic group underwent surgery at 2.7 ± 1.3 months after injury compared to the pre-pandemic cohort at 1.8 ± 1.1 months, which equates to an approximately four-week delay on average. Although time-to-surgery was significantly prolonged during the pandemic, the literature on surgical timing of ACLR suggests that this delay was insufficient to negatively impact outcomes after surgery or attainment of MCID.

Previous studies have investigated the impact of surgical timing and graft choice on pain following ACLR. While a delay in surgery greater than six months has been linked to more severe postoperative pain [18], other studies examining the impact of surgical timing on pain after ACLR have not found any significant associations [14,16]. In a randomized controlled trial including 69 patients, Bottoni et al. compared outcomes of early (less than 21 days after injury) versus delayed (greater than six weeks after injury) ACLR and found no difference in subjective evaluations of the knee [14]. Meighan et al. performed a similar randomized controlled trial including 31 patients and compared outcomes after surgery performed less than 14 days after injury to 8-12 weeks after injury and found no significant differences [16]. Regarding graft choice, a recent systematic review examining its effect on postoperative outcomes found that bone-patellar tendon-bone autografts (up to 72%) have a higher incidence of postoperative anterior knee and kneeling pain compared with hamstring (up to 44%) and quadriceps tendon autografts (up to 9.3%) [19].

As mentioned previously, ACLR was delayed by approximately six weeks in the pandemic group. Before and after matching, the use of bone-patellar tendon-bone allograft and quadriceps tendon autograft increased in the pandemic group while that of hamstring tendon decreased, likely reflecting decreased allograft availability during the pandemic and the recent trend favoring quadriceps tendon autograft [20]. When unmatched, our study demonstrated increased pain quantified by VAS scores at three months postoperatively and PROMIS-PI at six months postoperatively and final follow-up in the pandemic group. When matched, PROMIS-PI at six months postoperatively increased in the pandemic cohort and VAS scores at one year postoperatively increased in the pre-pandemic cohort. While the pre-pandemic and pandemic groups differed in levels of postoperative pain throughout the recovery period, the literature does not attribute these differences to surgical timing or graft choice. Also, the proportion of patients achieving MCID for PROMIS-PI at final follow-up was not significantly different between the cohorts, indicating that these differences were not of clinical importance.

Many prior studies have examined the effects of surgical timing on complications following ACLR and have reported variable findings. In a retrospective cohort study of 15,645 patients, Agarwal et al. found that a delay in ACLR of at least six weeks after injury was associated with a 65% risk reduction of operative intervention for arthrofibrosis in patients younger than 40 years [21-23]. In contrast, previous literature has shown that a delay in surgery is associated with a higher rate of meniscal tears and cartilage lesions [24-28]. Granan et al. estimated that the odds of a meniscal tear or cartilage lesion increased by 0.4% and 0.6% for each month that elapsed from injury to ACLR in a retrospective cohort study of 3,475 patients [26]. A meta-analysis of eight randomized controlled trials by Deabate et al. demonstrated that the risk of complication was not significantly different when comparing ACLR performed less than three weeks after injury to surgery performed greater than 10 weeks after injury [29]. Our study demonstrated that surgery was delayed by approximately four weeks during the pandemic. Despite this delay, no difference in complication rate was observed between the pre-pandemic and pandemic groups.

This study has a few limitations. Firstly, retrospective chart reviews rely on the accuracy and completeness of the information entered into the medical records from which data is extracted and hence are susceptible to biases inherently associated with retrospective study design. Second, due to the recency of the COVID-19 pandemic, this study could not report outcomes after ACLR beyond two years. Many important outcomes of ACLR can only be or are ideally observed over a longer follow-up period. For example, Pinczewski et al. reported that 25% of patients who undergo an ACLR will experience another tear in 10 years after their first injury [30]. Finally, although this study found no significant difference in several demographic factors, incidence of multi-ligamentous knee injury, and incidence of concomitant meniscal tears between the two groups, we did not measure several other factors that have been linked to outcomes after ACLR, such as psychological factors and preoperative level of activity [31,32].

## Conclusions

ACLR procedures at our institution were significantly delayed during the early months of the COVID-19 pandemic when compared to surgeries performed in 2018 (pre-pandemic era). While patients treated before and during the pandemic experienced varying levels of postoperative pain throughout the recovery period, there were no significant differences in functional outcomes or MCID achievement after surgery, nor any increased risk of complication or reoperation.
